# 

**Published:** 1889-06

**Authors:** 


A Brief Treatise on the Common Diseases of the Maxillary Sinus... 265
Copper Amalgam........ 282
MONTHLY DIGEST........ 292
A Bill for an Act to Regulate the Practice of Dentistry in the State of Minnesota........ 303

SELECTIONS.

Death from Chloroform in a Dentist's Chair........ 309
Prof. Winters on Constipation in Children........ 310
Over-Pressure in Children........ 311
Chicago Dental Society........ 311

EDITORIAL.

New Appliances........ 313
American Medical Association........ 313
A New Organization........ 314
Obituary........ 315

